# Adapting CRISPR/Cas9 System for Targeting Mitochondrial Genome

**DOI:** 10.3389/fgene.2021.627050

**Published:** 2021-04-06

**Authors:** Syed-Rehan A. Hussain, Mehmet E. Yalvac, Benedict Khoo, Sigrid Eckardt, K. John McLaughlin

**Affiliations:** ^1^Center for Molecular and Human Genetics, Abigail Wexner Research Institute, Nationwide Children’s Hospital, Columbus, OH, United States; ^2^Center for Clinical and Translational Research, Abigail Wexner Research Institute, Nationwide Children’s Hospital, Columbus, OH, United States; ^3^Department of Neurology, The Ohio State University Wexner Medical Center, Columbus, OH, United States

**Keywords:** mitochondria, heteroplasmic mutations, PNPase, RP-loop, chimeric guide RNA

## Abstract

Gene editing of the mitochondrial genome using the CRISPR-Cas9 system is highly challenging mainly due to sub-efficient delivery of guide RNA and Cas9 enzyme complexes into the mitochondria. In this study, we were able to perform gene editing in the mitochondrial DNA by appending an NADH-ubiquinone oxidoreductase chain 4 (ND4) targeting guide RNA to an RNA transport-derived stem loop element (RP-loop) and expressing the Cas9 enzyme with a preceding mitochondrial localization sequence. We observe mitochondrial colocalization of RP-loop gRNA and a marked reduction of ND4 expression in the cells carrying a 11205G variant in their ND4 sequence coincidently decreasing the mtDNA levels. This proof-of-concept study suggests that a stem-loop element added sgRNA can be transported to the mitochondria and functionally interact with Cas9 to mediate sequence-specific mtDNA cleavage. Using this novel approach to target the mtDNA, our results provide further evidence that CRISPR-Cas9-mediated gene editing might potentially be used to treat mitochondrial-related diseases.

## Introduction

Mitochondrial diseases caused by defects in mitochondrial oxidative phosphorylation (OXPHOS) can be linked to the presence of wild type and mutant variants of mitochondrial genes in the cells, which is called heteroplasmy. Dependent on levels of heteroplasmy, they can manifest as highly heterogeneous disorders resulting in phenotypes ranging from mild hearing impairment to severe progressive multisystem disorders (Wallace, 2010; Dhillon and Fenech, 2014; Lightowlers et al., 2015). Genetically, mitochondrial disorders typically include those resulting from variants in the mitochondrial genome (mtDNA) or those associated with alterations in nuclear genes coding for mitochondrial proteins. As many as 1:200 individuals harbor a pathogenic mtDNA variant that could be transmitted to the offspring (Elliott et al., 2008), and the population prevalence for specific pathogenic mtDNA variants is as high as 1:400 in diseases such as mitochondrial encephalomyopathy, lactic acidosis, and stroke-like episodes (MELAS 3243A > G mtDNA mutation) (Manwaring et al., 2007; Grady et al., 2018). In most disorders associated with variants in mtDNA, the individuals’ cells are heteroplasmic in that they contain a mixture of a variant and wild-type mitochondrial genomes (wt-mtDNA), with ratios that can also be tissue specific. The manifestation of defects associated with pathogenic mtDNA variants and the severity of symptoms depends on reaching of the proportion of variant mitochondria to certain threshold levels, which is related to both nature of the variant and to tissue-specific energy expenditure relying on mitochondria (Wallace and Chalkia, 2013). Strategies to prevent transmission of mtDNA disorders include the use of donated oocytes, preimplantation genetic diagnosis, and mitochondrial replacement therapy (Craven et al., 2010; Wolf et al., 2015; Falk et al., 2016), which involves the transfer of nuclear DNA from a heteroplasmic oocyte or embryo into a donor cytoplast with wild-type mitochondria by pronuclear or spindle transfer (Wolf et al., 2015). Although successful in humans, recent data have shown that incompatibility between donor and host mitochondria is associated with genetic drift leading to loss of donor mtDNA and reversion to the mutant haplotype (Kang et al., 2016; Yamada et al., 2016). An alternative approach to reduce heteroplasmy for variant mtDNA below threshold levels could be the use of mitochondria targeting nucleases that selectively cleave specific mtDNA haplotypes, resulting in their degradation and shifting the heteroplasmy ratio toward the wt-mtDNA haplotype. Proof-of-principle for this concept has been demonstrated with restriction endonucleases (Patananan et al., 2016), mitochondrial zinc-finger nucleases (mtZFN) (Gammage et al., 2014, 2016), and mitochondrial transcription activator-like effector nucleases (mito-TALENs) (Bacman et al., 2013; Hashimoto et al., 2015; Reddy et al., 2015). In contrast to restriction enzymes for which only a few applicable mutant sequences exist, mtZFN and mito-TALENs can be designed and engineered to selectively cleave a range of mtDNA sequences (Bacman et al., 2014; Gammage et al., 2016; Phillips et al., 2017). Limitations to these approaches include the relatively labor and cost-intensive production, and achieving size compatibility with current virus-based delivery systems to tissues, and the need for repeated transfections to achieve an effective heteroplasmy shift (Hashimoto et al., 2015; Gammage et al., 2016). Another powerful genome editing and targeting methodology is based on clustered regularly interspaced short palindromic repeats (CRISPR), which is a part of bacterial immune systems that recognizes a 2- to 6-bp DNA sequence called protospacer adjacent motif (PAM) immediately following the DNA sequence targeted by a nuclease (Wang et al., 2016). The most widely used constructs use the *CRISPR*-associated protein-9 nuclease (*Cas9*) from *Streptococcus pyogenes* (spCas9) and a chimeric single-guide RNA (sgRNA) recognizing NGG as a PAM sequence. Development of this and other emerging CRISPR-based gRNA editing tools such as the Cpf1-family T-rich PAM sequence at the 5′ end compared with the spCas9 recognition sequence 3′-NGG (Zetsche et al., 2015) could be very effective in editing mitochondrial DNA. However, a major obstacle in the development of CRISPR-Cas9-mediated gene editing of the mitochondrial genome is the lack of effective methods to deliver gRNA through the mitochondrial membrane since the import of mRNAs into the mitochondria is largely dependent on tRNA (Kolesnikova et al., 2004; Karicheva et al., 2011; Gammage et al., 2018).

One approach to deliver nuclear-encoded RNA into the mitochondria involves the utilization of a native RNA transport enzyme, polynucleotide phosphorylase (PNPase) encoded by *PNPT1* gene, located in the mitochondrial intermembranous space (Wang et al., 2012). There is increasing evidence of PNPase involvement in the import of small RNAs, into mitochondria (Shepherd et al., 2017; Sato et al., 2018). Furthermore, it has been shown that the addition of the 20 nucleotide stem-loop sequence of nuclear RNAse P to the transcripts not normally heading to the mitochondria facilitates transportation of these RNAs into the mitochondria in a PNPase-dependent manner (Wang et al., 2010). However, this approach has not yet been demonstrated for the delivery of sgRNAs into the mitochondria.

To overcome the challenges of delivering gene-editing tools and facilitate gene deletion for specific mitochondrial diseases, we have developed a novel hybrid guide RNA construct (sgRNA) in which the 20-nucleotide RNA stem-loop structure of RNAse P was appended at the 5′ end of the sgRNA sequence. Here, we hypothesized that PNPase-dependent RNA import can be used to transport hybrid sgRNA into the mitochondria and that concurrent expression of Cas9 in the mitochondria through the delivery of Cas9 mRNA with a mitochondria localization signal would result in sequence-specific cleavage of mtDNA in the cell that will be able reduce the heteroplasmy in mtDNA disorders. We designed a hybrid sgRNA specific to the 11205G region in of the mtDNA-encoded mouse mitochondrial-encoded NADH:ubiquinone oxidoreductase core subunit 4 (mtND4) gene and incorporating the RNAse P stem-loop sequence and co-transfected this construct into the cells along with mitochondria-targeting Cas9 constructs (MLS-Cas9). The results showed that the population of mtDNA carrying 11205G in their mtND4 sequence was reduced remarkably, and ND4 expression in the cells was decreased. Furthermore, a fluorescent-tagged gRNA sequence with RP-loop colocalized with the mitochondria. This suggests that CRISPR/Cas9 targeting of the mitochondria utilizing PNPase-mediated transport is feasible and promise a new opportunity to eliminate disease-causing mutations in the mitochondrial genome.

## Materials and Methods

### CRISPR/Cas9 Constructs

The expression construct pX330-U6-Chimeric_BB-CBh-hSpCas9 (Addgene; plasmid # 42230 (Cong et al., 2013) for human codon-optimized SpCas9 and a chimeric guide RNA was modified as follows: (i) the two nuclear localization signals (NLS) flanking the N and C terminals of Cas9 were replaced with two mitochondrial localization signals (MLS1 and MLS2); and (ii) the human SpCas9 sequence was codon optimized for mouse expression (in the following referred to as mSpCas9). This modified expression plasmid is referred to as pX-U6-chimeric-MLS-mSpCas9. MLS1 consists of the amino-terminal leader peptide of mouse ornithine transcarbamylase (Seibel et al., 1995), and MLS 2 is of the 23-amino acid leader peptide of cytochrome oxidase subunit 8 (COX 8; (Bacman et al., 2013)). The mSpCas9-MLS construct was synthesized by GenScript (NJ, United States) using their OptimumGene^TM^ – Codon Optimization algorithm.

**FIGURE 1:**
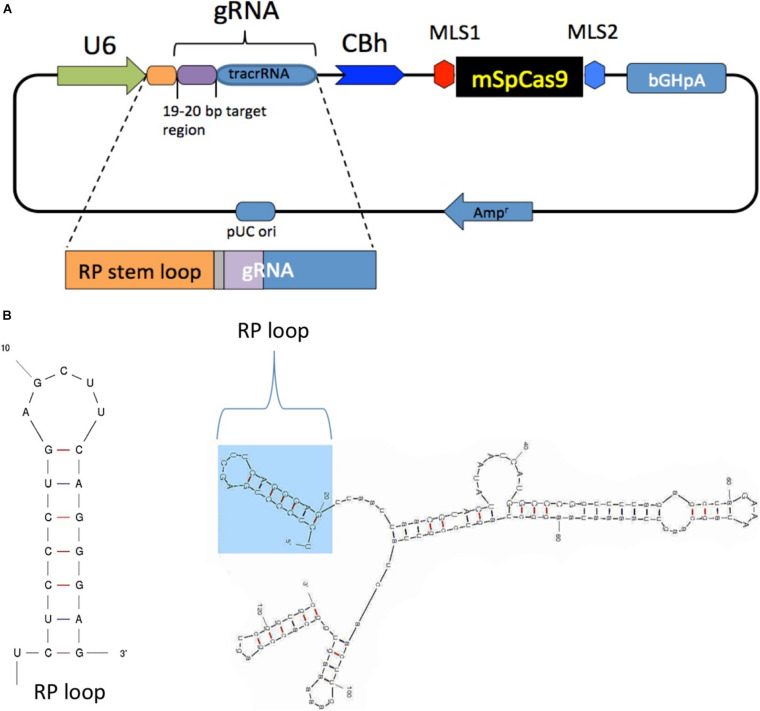
Generation and structure of mitochondria targeting the clustered regularly interspaced short palindromic repeats (CRISPR)–*CRISPR*-associated protein-9 nuclease (Cas9) complex. **(A)** Mitochondrial RP loop and guide RNA (gRNA) targeting ND4 is in the downstream of U6 promoter, and chicken beta actin (CBh) controls the expression of mSpCas9 flanked by MLS1 and MLS2 sequences. **(B)** M-Fold analysis of chimeric gRNA with RP loop.

The chimeric guide RNA was constructed as follows: a CRISPR design online tool^1^ was used to select 19- to 20-bp target regions in the mouse mitochondrial-encoded NADH:ubiquinone oxidoreductase core subunit 4 (mtND4) with target nucleotide G11205 that is in a natural PAM sequence starting at position 11,204 (AGG). The selected sgRNA sequence with an additional 20-bp RP-loop (5′TCTCCCTGAGCTTCAGGGAG-3′) at the 5′ end of guide RNA was custom synthesized by GenScript, cloned into plasmid pUC57 with unique restriction sites (Pcil, Xba1), and then sub-cloned into pX-U6-chimeric-MLS-mSpCas9 to generate pX-U6-RP-sgRNA-MLS-Sp Cas9 (Figure 1A). The same vector without the RP-loop sequence (pX-U6-sgRNA-MLS-mSpCas9) was used as the control. Both constructs were then used as templates for *in vitro* RNA synthesis of sgRNAs with or without RP loop or Cas9 with MLS. RNA secondary structure predictions for RP Loop CRISPR were performed using *M-fold* software for recombinant sgRNA modeling (Figure 1B; Zuker, 2003).

### Generation of MLS-Cas9 mRNA and RP Loop-sgRNA

The DNA template for *in vitro* transcription of mSpCas9 was generated by PCR amplification of pX-U6-RP-sgRNA-MLS-mSpCas9, using a forward primer that included a T7 promoter (T7 HAtag-Cas9-F: 5′TAATACGACTCACTATAGGGATGTAC CCATACGATGTTCCAGATTACGCT-3′) and a reverse primer (Cas9-R: 5′-GCGAGCTCTAGGAATTCTTAC-3′). Cas9 mRNA (referred to as Cas9 in the following) was then synthesized, using the mMESSAGE mMACHINE^®^ T7 Ultra Kit (Life Technologies, Carlsbad, CA, United States) and purified by lithium chloride precipitation. *In vitro* RNA synthesis of sgRNA with and without RP loop was also performed by using DNA templates of sgRNAs generated by PCR amplification of plasmid constructs, using construct-specific forward primers that included a T7 promoter (pX-U6-RP-sgRNA-MLS-Sp Cas9, T7-RPloop-F: 5′TAATACGACTCACTATAGGGTCTCC CTGAGCTTCAGGGAGT-3′; pX-U6-sgRNA-MLS-Sp Cas9, T7-NoLoop-F: 5′TTAATACGACTCACTATAGGGCGTACTATAAT CATGGCCCG-3′) and a common reverse primer (sgRNA-R: 5′-AAAAGCACCGACTCGGTGCC-3′). The RP loop sgRNAs were then synthesized using the MEGAshort-script^TM^ T7 Kit (Life Technologies). RNA was purified and concentrated by using RNA Clean & Concentrator-5 Kit (Zymo Research Corp., Irvine, CA, United States). The integrity of the synthesized RNAs was assessed using Agilent RNA 6000 Nano Kit with Agilent 2100 Bioanalyzer (Agilent Technologies, Santa Clara, CA, United States). These newly synthesized sgRNA constructs will be referred to as RP Loop sgRNA (RP-loop sgRNA) and No RP Loop sgRNA (sgRNA).

### Cell Culture

Primary mouse embryonic fibroblast (MEF) were derived from Tg(DR4) 1Jae/J mice stock No: 003208 (Jackson Laboratories). Human HEK293K cells were ATCC CRL-1573 (American Type Culture Collection, United States). Transient transfection with synthetic mRNA of Cas9 and sgRNAs was performed in either MEF or 293K cells using the TransIT^®^-mRNA Transfection Kit (Mirus Bio LLC) in OptiMEM medium (Invitrogen). For experiments with only wild-type mtND4 MEFs being transfected with the CRISPR/Cas9 system (all except those in Figure 3A), DMEM medium was supplemented with 50 μg/ml of uridine and 100 mM pyruvate to improve cell survival after transfections.

### *In vitro* Assay to Test Mito-CRISPR Functionality and Specificity

Mito CRISPR target selection was performed using the GeneArt Genomic Cleavage Selection Kit (cat# A27663 Thermo Fisher Scientific), which is based on restoration of reporter Orange Fluorescent Protein (OFP) expression if endonuclease activity at a target sequence induces DNA double-stranded break and repair. Briefly, following the manufacturer’s protocol, 23-nt single-stranded DNA sequences of the mtND4 target sequence, containing either the target nucleotide (11205G) or non-target control (11205A) at the PAM sequence, were synthesized, converted into double-stranded oligonucleotide of each variant, and duplex oligonucleotides were separately cloned into the pGCS reporter vector producing pGCS-wt (11205G) and pGCS-variant (11205A). Cotransfection of pX-U6-RP-sgRNA-MLS-Sp Cas9 with reporter constructs into HEK293K was performed using TransIT^®^-2020 transfection reagent (Mirus Bio LLC). Orange fluorescence, indicative of genomic cleavage by CRIPR/Cas9 complex, was visually assessed at 48–72 h posttransfection using an EVOS cell imaging system (Thermo Fisher Scientific, United States).

### Immunostaining

Mouse embryonic fibroblast cells were grown on coverslips in 12-well plates and transfected with either pX330-U6-Chimeric_BB-CBh-hSpCas9 or pX-U6-chimeric-MLS-mSpCas9. After 24 h, the medium was removed, and cells were fixed by two brief washes in ice-cold acetone. Cells were blocked in 3% goat serum in 1x TBS for 30 min, followed by immunostaining with Cas9 monoclonal antibody 4G10 (Diagenode cat#: C15200216; 1:200 dilution) with gentle shaking for 2 h at room temperature. Cells were washed in 1% goat serum in 1x TBS, followed by incubation with a secondary anti-mouse antibody conjugated with Alexa 488 (Molecular Probes) 1:1,000 dilution for 1 h at room temperature. Cells were washed with 1.0% goat serum in 1x TBS cells and mounted in Vectashield with DAPI, and images were taken using a confocal microscope (Zeiss LSM 700).

### Fluorescent Labeling and Mitochondrial Tracking of Guide RNA

RP-loop sgRNA and sgRNA without RP-loop, labeled with Alexa flour 488 at the 3′ end was custom synthesized by IDT Integrated DNA Technologies, Inc. Due to size limitations for the synthesis of fluorescently labeled RNA, the tracrRNA sequence were truncated at the 3′ end to the following sequences: guide RNA with RP-loop as RP-ND4crRNA (sequence: 5′rUrCrUrCrCrCr UrGrArGrCrUrUrCrArGrGrGrArGrUrUrArArUrUrArArCrGr UrArCrUrArUrArArUrCrArUrGrGrCrCrCrGrGrUrUrUrUrAr GrArGrCrUrArGrArArA/3AlexF488N/) and without RP-loop as ND4crRNA (5′rUrUrArArUrUrArArCrGrUrArCrUrArUrAr ArUrCrArUrGrGrCrCrCrGrGrUrUrUrUrArGrArGrCrUrArGr ArArA/3AlexF488N/). RP-ND4crRNA and ND4crRNA were transfected into MEF cells. The mitochondria in live MEF were stained at 18 h posttransfection with 500 nM of MitoTracker Deep Red FM (Cat# M22426, Invitrogen) at 37°C with 5% CO_2_ for 30 min. Images were captured on a motorized Nikon Ti2-E inverted microscope with a 100x Plan Apochromat Lambda oil immersion objective and a Hamamatsu ORCA Fusion camera within 2 h of completion of staining. The final images had a resolution of 0.064 μm/pixel and were processed in an automated and identical fashion in NIS-Elements AR software (Nikon Instruments) using the denoising and Clarify.ai (a deep learning image processing tool trained to remove out-of-focus fluorescence signal from widefield microscope images to improve the signal-to-noise ratio and resolve structural features). After processing, identical brightness, and contrast adjustments were applied to all images to improve visibility of defining features.

### Mitochondrial DNA Isolation

Mitochondrial DNA was isolated using Mitochondrial/Cytosolic Fractionation Kit (BioVision Inc., CA, United States; cat# K256-25) according to the manufacturer’s instructions. MEF cells were grown in six-well plates to 80% confluency before transfection with RP-sgRNA11205G (1.5 μg/well) using TransIT-mRNA Transfection Kit (Mirus Bio LLC). After 24 h of posttransfection, 5 × 10^6^ cells were harvested using trypsin (0.05% trypsin-EDTA). Cell membranes were disrupted in cytosolic buffer using a Dounce homogenizer followed by successive centrifugation steps at 700 × *g* to collect supernatant followed by 10,000 × *g* centrifugation to collect intact mitochondria. Mitochondrial pellets were then used for isolating mito-DNA using a QIAamp DNA mini Kit (Qiagen). DNA was eluted in water and quantified by NanoDrop 2000 UV-Vis Spectrophotometer (Thermo Fisher Scientific).

### Quantitative PCR

DNA and RNA were isolated using QIAamp DNA Mini Kit (Qiagen) and mirVana RNA isolation kit (Ambion-Thermo Fisher Scientific), respectively. cDNA synthesis of RNA was performed by using SuperScript^®^ VILO^TM^ cDNA Synthesis Kit (Thermo Fisher Scientific; cat# 11754050). QPCR was performed using Precision Melt Supermix containing EvaGreen dye (cat# 172-5110) using CFX96 Touch^TM^ Real-Time PCR Detection System (Bio-Rad, United States). Sequences of PCR primers are mND4-F: AGT TAG CCA CAT AGC ACT TGT; mND4-R: GCT AGA CTT GCT ATC AGT CAT; mCox3-F: GAA ACC ACA TAA ATC AAG CCC TAC; mCox3-R: GTT GTC GTA GGC AAA CAA TAA G; mND1-F: TCC TAA CAC TCC TCG TCC CC; mND1-R: TGG CGT CTG CAA ATG GTT GT; mSdhA-F: TAC TAC AGC CCC AAG TCT; mSdhA-R: TGG ACC CAT CTT CTA TGC; RPloop-F: CCT GAG CTT CAG GGA GTT AAT; RP loop-R: CGA CTC GGT GCC ACT TTT TC.

## Results

### mSpCas9 Does Not Localize to the Nucleus

**FIGURE 2:**
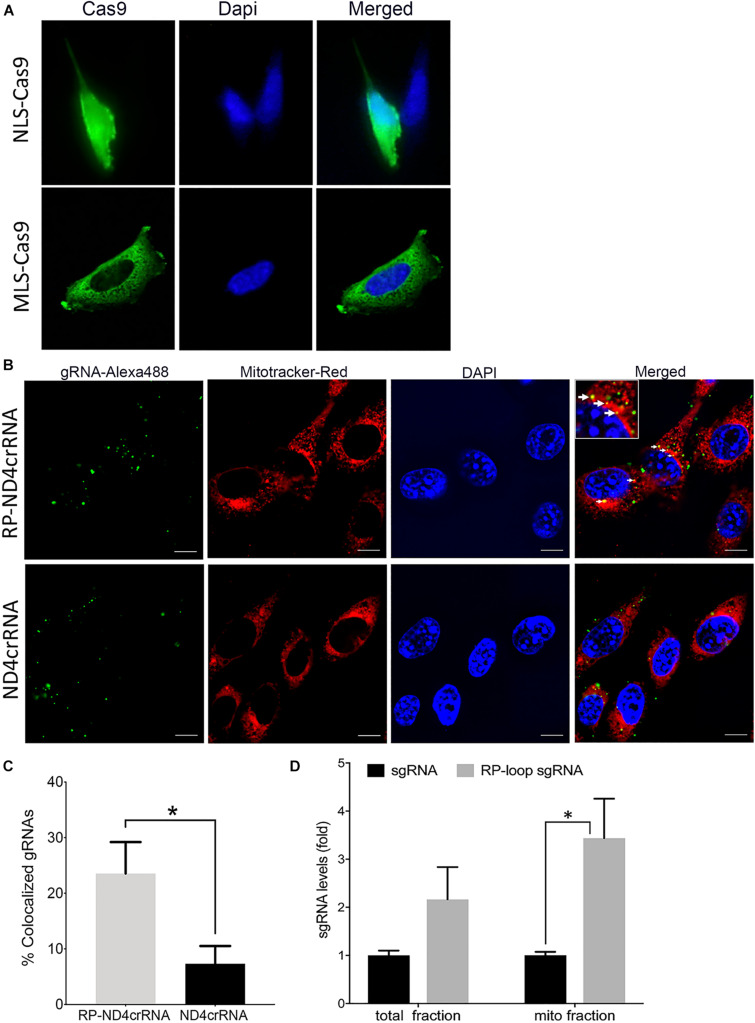
Delivery of CRISPR–Cas9 complex into the mitochondria. **(A)** Immuno-staining showed that at 24 h posttransfection, mitochondria-targeting Cas9 constructs (MLS)–Cas9 predominantly localized in the cytoplasm, whereas nuclear localization signal (NLS)–Cas9 went to the nucleus. **(B)** Mouse embryonic fibroblast (MEF) cells were transfected with Alexa flour 488 tagged ND4crRNA and RP-ND4crRNA, and the mitochondria were stained after 18 h with MitoTracker^®^ Deep Red FM. Cells were mounted in Vectashield anti-fade mounting media with DAPI (Vector Laboratories), and images were taken at 100× magnifications using Nikon Ti2-E inverted microscope; scale bar: 10 μm. **(C)** Cells with mitochondrial colocalized gRNA (solid arrows in **B**) were counted at 20× magnification showed enhanced percentage of RP-ND4crRNA merged with mitochondria (*n* = 3, Student’s *t*-test, *p* = 0.01). **(D)** RP loop increases the levels of sgRNA detected in the mito fractions. qPCR was performed, and levels of sgRNA were normalized to mitochondrial ND4 and ND1 as internal controls and reported as fold change 2^ΔΔ*CT*^ (*n* = 3, Student’s *t*-test, *p < 0.05).

To enable transport of Cas9 into the mitochondria of mouse cells, we modified a CRISPR/Cas9 expression plasmid (pX330-U6-Chimeric_BB-CBh-hSpCas9 (Cong et al., 2013) such that a mouse-optimized Cas9 sequence was flanked by two mitochondrial localization signals (MLS) (Figure 1A). The amino terminal signal (MLS1) consisted of the amino-terminal leader peptide of mouse ornithine transcarbamylase (Seibel et al., 1995), and the C-terminal MLS2 consisted of the 23-amino acid leader peptide of cytochrome oxidase subunit 8 (Bacman et al., 2013). Because codon bias can affect translation and activity of Cas9 protein in cell culture systems (Weninger et al., 2016), we also used a Cas9 coding sequence that was optimized for mouse expression. As expected, transfection of MEF cells with pX330-U6-Chimeric_BB-CBh-hSpCas9 (Cong et al., 2013), in which the coding sequence of Cas9 is flanked by nuclear localization signals, resulted in a strong nuclear signal of Cas9 in immunostaining assays (Figure 2A, top panel). In contrast, cells transfected with the modified expression construct encoding Cas9 flanked by MLS1 and MLS2 lacked Cas9 signal in the nuclei and exhibited immunostaining throughout the cytoplasm. We did not perform mitochondrial staining for co-localization as the two leader peptides used in our construct are well known for delivery into the mitochondria, and the objective of performing Cas9 IHC was only to confirm that by replacing NLS with MLS, we have indeed prevented nuclear translocation of this modified Cas9 suggesting mitochondrial localization (Figure 2A, bottom panel).

### Guide RNA Delivery Into the Mitochondria

To determine whether RP loop mediates import of guide RNA (sgRNA) constructs into the mitochondria, we incorporated a 20-nucleotide stem-loop element (RP-loop) that is a component of nuclear RNAse P at the 5′ end of guide. PNPase is localized to the inner mitochondrial membrane and regulates the import of nuclear-encoded RNAs into the mitochondrial matrix. Addition of the RP-loop to transcripts that do not normally translocate to the mitochondria has been shown to allow for RNA import in a PNPASE-dependent manner (Wang et al., 2010). Furthermore, this delivery system has been previously reported to mediate the targeted transfer of recombinant RNAs into the mitochondria (Wang et al., 2010; Comte et al., 2013). We therefore constructed a hybrid sgRNA in which the RP loop was appended to the 5′-end of the sgRNA11205G construct designed to selectively base pair with the wild-type mtND4 target −20 nucleotide sequence. To facilitate future sub-cloning, an eight-nucleotide Pac1 restriction site separated the RP-loop and sgRNA (Figure 1A). Based on structure predictions using the M-fold algorithm, the hybrid RP-loop-gRNA maintained the secondary structure of the stem-loop required for mitochondrial import (Initial ΔG = −42.60 kcal/mol at 37°C) (Figure 1B).

To test if the RP-loop sequence facilitated sgRNA localization to the mitochondria, we transfected MEF cells with an Alexa flour 488-labeled RNA containing the stem-loop (RP-ND4crRNA) and with a control RNA without stem-loop (ND4crRNA). RP-ND4crRNA-transfected cells contained a significantly higher number of cells in which fluorescent RNA signal colocalized with the mitochondria than ND4crRNA-transfected cells (*p* < 0.01) (Figures 2B,C).

As additional evidence of mitochondrial localization of the RP-loop sgRNA construct, we transfected RP-loop sgRNA11205G and sgRNA11205G into MEF cells and isolated mitochondrial fractions after 24 h qPCR showed that though both full-length RP-loop guide RNA and sgRNA were present in the mitochondrial fraction, RP-loop sgRNA was highly enriched in the mitochondrial fraction of transfected cells versus sgRNA controls (Figure 2D) indicating that RP loop-added sgRNAs are efficiently transported into the mitochondria. The presence of sgRNA without RP loop in the mitochondrial fraction may be due to unregulated transport between inner and outer mitochondrial membrane that has earlier been proposed to facilitate CRISPR/Cas9-based editing in the mitochondria (Jo et al., 2015).

### Targeting Mitochondrial DNA Using CRISPR/Cas9

To evaluate CRISPR/Cas9-mediated cleavage of a mitochondrial-encoded gene, we used mouse mitochondrial-encoded NADH:ubiquinone oxidoreductase core subunit 4 (mtND4). A previous study demonstrated that mouse mtDNA variants with relevance to human diseases can be generated in mouse cell lines. For example, a G11186A variant in mtND4 sequence corresponds to a mutation at nucleotide 11,795 of the human mtDNA causing mtND4-associated complex I deficiency and respiration defects (Fayzulin et al., 2015).

Here, as a model to demonstrate mutation-specific selective cleavage of mtDNA sequences, we used mtND4 variants at position 11,205 (G/A). Effectively, this approach would be useful to enrich for a variant in a heteroplasmic cell by eliminating or reducing the fraction corresponding to the disease-causing variant. Using mouse mtDNA sequences, we selected target guide sequences against base 11,205G of mt-ND4 and used these to construct a hybrid guide RNA (sgRNA11205G) composed of the CRISPR array and tracrRNAs (Cong et al., 2013). The guide sequence selected (5′CGTACTATAATCATGGCCCG-3′) scored 92 on the scale of 0–100 to indicate the faithful on-target activity of guide with only one off-target site.

**FIGURE 3:**
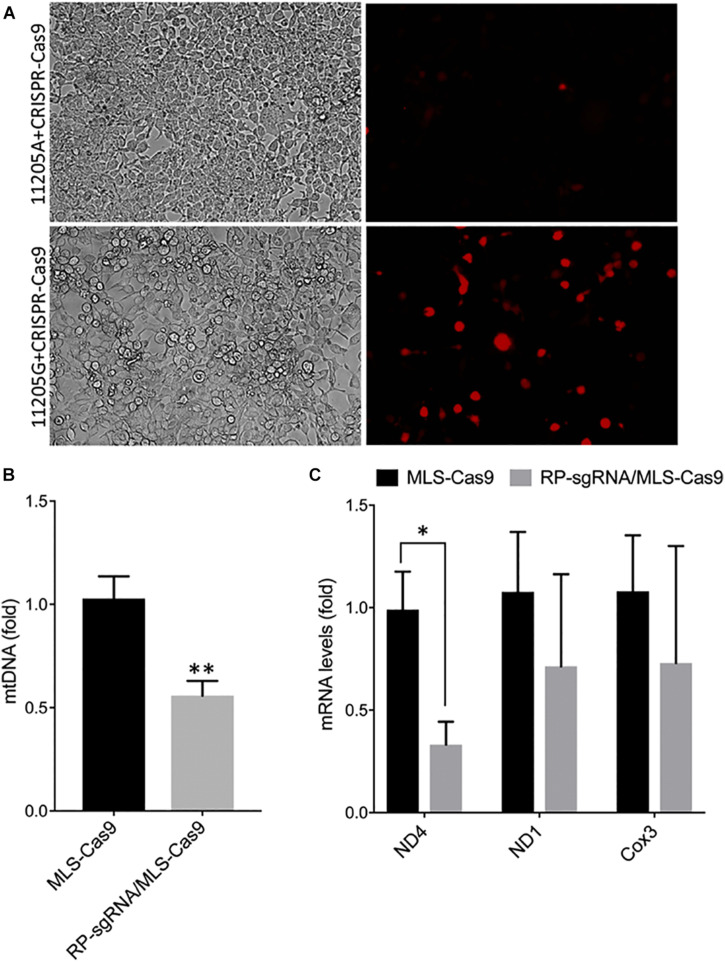
Specificity and functionality of RP loop CRISPR/Cas9 complex. **(A)** Specificity of RP loop-CRSPR-/Cas9 was determined using the GeneArt^®^ Genomic Cleavage Selection Kit that expressed orange fluorescent reporter, in 293K cells 72 h posttransfection, specific to the 11205G nucleotide of ND4. qPCR for mtDNA levels. **(B)** qRT-PCR for ND4 mRNA expression **(C)** was significantly lower in RP loop sgRNA-CRISPR/Cas9-transfected MEF cells, at 24 h posttransfection, compared with the MLS–Cas9 controls (*n* = 5, Student’s *t*-test, *p = 0.016; **p = 0.005).

The activity and sequence-specificity of the hybrid sgRNA construct incorporating the RP loop at the 5′ of the guide sequence was verified by an *in vitro* assay for genomic cleavage as described in the “Materials and Methods” section. We observed OFP fluorescence only in cells transfected with 11205G reporter constructs but not 11,205A constructs, indicating base-specific cleavage of the 11205G sequence (Figure 3A). This *in vitro* assay further demonstrates that the RP loop sgRNA is both functional and sequence specific, promoting cleaving of mtDNA by Cas9 endonuclease.

### Inclusion of the Stem Loop Facilitates CRISPR-Cas9-Mediated Reduction of mtDNA With A11205G Variant

With the inclusion of MLS sequences, Cas9 can be translocated to the mitochondria. However, functional localization within the mitochondria requires evidence of targeted mtDNA endonuclease activity. To address this, we cotransfected wild-type MEFs with an MLS-Cas9 expression construct either as mRNA or as plasmid alone or in combination with RP-sgRNA11205G. The CRISPR/Cas9 complex with RP-loop sgRNA11205G was able to significantly reduce mtDNA levels relative to Cas9 alone (*P* = 0.005; Figure 3B). This knockdown of mtDNA levels post 24 h by MLS-Cas9 and RP-sgRNA11205G was significant when compared with the cells treated with MLS-Cas9 alone. To ascertain whether mtDNA knockdown downregulates ND4 gene expression, we next quantified expression levels of mitochondrial-encoded transcripts in wild-type MEF with 11205G in the mtND4 sequence. At 24 h posttransfection, we observed a significant reduction of ND4 transcripts in the RP-sgRNA11205G cells vs. cells transfected with sgRNA11205G, using GAPDH as an internal nuclear RNA control (Figure 3C). Other mitochondrial genes such as ND1 and Cox3 also had lower transcripts levels in RP-sgRNA11205G-transfected cells, but the reduction was not significant vs. control-transfected cells. Therefore, the lower level of these transcripts may reflect the lower levels of mtDNA overall due to CRISPR/Cas9-mediated cleavage of the mitochondrial genome (Figure 3C) that has been shown to rapidly degrade *in vivo* (Moretton et al., 2017; Peeva et al., 2018).

## Discussion

Our objective was to determine whether it was possible to enable or enhance CRISPR/Cas9 mitochondrial genome editing utilizing PNPASE, a native RNA transport system, although the mechanism of augmented import of RNA by PNPASE is not fully understood (Patananan et al., 2016). We conclude that both the targeted mtDNA and its transcripts levels are reduced and correlate with the designed mitochondrial import of the target-specific guide RNA. Previous reports of CRISPR-based editing (Zetsche et al., 2015) have shown similar levels of reduction in targeted mtDNA and transcripts without modifying the gRNA; however, we only observe this in our modified sgRNA specific for the target sequence allowing uptake into the mitochondria via PNPase. The differences may be attributed to unregulated uptake of small RNA into the mitochondria or the difference in transfection method, incubation time, and repeated transfections. One of the critical experiment for the proof-of-concept was to visually verify that sgRNA can be imported into the mitochondria if an RP loop is appended. We selected Alexa flour 488 to label the construct because, unlike other fluorescent dyes such as Cy3, this dye does not accumulate on the mitochondrial surface (Rhee and Bao, 2010). Therefore, we posit that if RP loop is guiding the RNA, then visualization of Alexa Flour 488-labeled gRNA to the mitochondria would be possible. Our results show an enhanced delivery of sgRNA with RP loop into the mitochondria. A recent study has shown that guide RNA with short hairpin structures can promote mitochondrial import and specific cleavage of mtDNA albeit at a low level due to limited import into the mitochondrial matrix (Loutre et al., 2018a, b). This study further strengthens our hypothesis that specific hairpin structures that are involved in the delivery of nuclear-encoded RNA can serve as potential adapter for delivery, and PNPase may serve as a conduit for channeling this recombinant RNA into the mitochondrial matrix. The presence of multiple mitochondrial genomes in each cell may result in only a cleavage of a subset of mitochondrial genomes with variable impact on viability of each mitochondrion. Presumably repeated treatment or longer exposure times would correspond with an increase in mitophagy in competition with replication of non-targeted mitochondrial genomes. Furthermore, a rapid reduction in mtDNA copy number as shown with previous studies using mito-TALEN creates potential risk of developing mtDNA depletion syndrome, which limits the clinical implication (Hirano et al., 2018).

For all our studies with knockdown, the DNA or RNA was isolated 24 h after transfection, at a point that we found an optimal choice for loss of cell viability and mitochondrial gene expression. To avoid cell death, we used *in vitro* synthesized RNAs for both sgRNA and Cas9 instead of using plasmid, which would continuously express uncontrolled levels of the complex. Moreover, this approach only reduces ∼25% of mtDNA, which would avoid any mitochondrial depletion syndrome. Our present study provides support for the function of the RP loop as an enhanced mitochondrial transporter of gRNA for CRISPR-Cas9 machinery across the mitochondrial membranes.

Currently, validation of alternative endonuclease-based systems, including TALENs and ZFN, is ongoing in clinical trials and presently have the advantage of a large range of targetable nucleotides within the mitochondrial genome. Furthermore, CRISPR-free mitochondrial editing has recently been reported using an interbacterial cytidine deaminase fused to the TALE protein used in TALENs. Though the approach to base substitute CG to TA in mtDNA is promising, to our knowledge and as reported by others, there is only one human point mutation that can use this approach (Mok et al., 2020; Reddy et al., 2020). Moreover, it cannot be effective in editing when there is large-scale deleted mtDNA as in Pearson and Kearns–Sayre syndromes.

The primary appeal for using CRISPR/Cas9 endonuclease is ease of design and speed of execution. With the introduction of other variants of Cas9 such as saCas9, Cpf1, which performs cutting functions analogous to spCas9 but have different PAM recognition sequence and smaller size of endonuclease, there will be more efficient delivery to the cells and the mitochondria. We anticipate that our proof-of-concept study with chimeric guide RNA with RP loop may be also able to deliver other CRISPRs to the mitochondria. This will increase the available target sequences in the mitochondrial genome for potential therapeutic option by shifting heteroplasmy.

**FIGURE 4:**
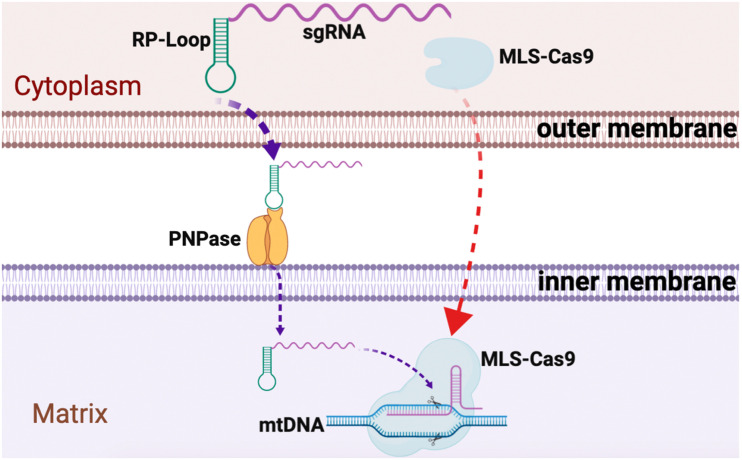
Graphical summary of CRISPR/Cas9 delivery into the mitochondria. Guide RNA, with RP loop attached upon transfection, is imported into the mitochondria by attaching to PNPase in the inner mitochondrial membrane (IM). Cas9 protein with N-terminus mitochondria localization sequences (MLS) targets Cas9 to the mitochondria where it forms a complex with the RP loop sgRNA to cause sequence-specific mtDNA cleavage.

We observed relatively robust RP loop-mediated mitochondrial localization of sgRNA in the mitochondria potentially mediated via PNPase present in the inner mitochondrial membrane, and with MLS-Cas9 in the mitochondria, the CRISPR/Cas9 complex promoted mtDNA knockdown (Figure 4). The utility of the RP loop-mediated RNA transport versus other modes is only relevant if the engineered heteroplasmic shift is sufficient to address the symptomatic manifestation of mitochondrial diseases. In subsequent studies, we plan to examine our mito-CRISPR/Cas9 tools in human cybrid cells with heteroplasmy such as 3243A > G, a common mutation in MELAS, and will test the efficiency of adeno-associated viral vector (AAV) to carry mito-CRISPR/Cas9 to eliminate mutant mito-DNA.

## Data Availability Statement

The raw data supporting the conclusions of this article will be made available by the authors, without undue reservation.

## Author Contributions

S-RAH and KJM are co-senior authors and have conceptualized the project, designed the research, and wrote the manuscript. S-RAH and BK performed the experiments. MEY and SE contributed to the writing and discussion. All authors approved the manuscript.

## Conflict of Interest

The authors declare that the research was conducted in the absence of any commercial or financial relationships that could be construed as a potential conflict of interest.

## Footnotes

1 http://crispr.mit.edu/guides

## References

[B1] Bacman S. Williams S. Pinto M. Moraes C. (2014). The use of mitochondria-targeted endonucleases to manipulate mtDNA. *Methods Enzymol.* 547 373–397. 10.1016/b978-0-12-801415-8.00018-7 25416366 PMC4274129

[B2] Bacman S. Williams S. Pinto M. Peralta S. Moraes C. (2013). Specific elimination of mutant mitochondrial genomes in patient-derived cells by mitoTALENs. *Nat. Med.* 19 1111–1113. 10.1038/nm.3261 23913125 PMC4153471

[B3] Comte C. Tonin Y. Heckel-Mager A. Boucheham A. Smirnov A. Aure K. (2013). Mitochondrial targeting of recombinant RNAs modulates the level of a heteroplasmic mutation in human mitochondrial DNA associated with Kearns Sayre Syndrome. *Nucleic Acids Res.* 41 418–433. 10.1093/nar/gks965 23087375 PMC3592399

[B4] Cong L. Ran F. Cox D. Lin S. Barretto R. Habib N. (2013). Multiplex genome engineering using CRISPR/Cas systems. *Science* 339 819–823. 23287718 10.1126/science.1231143 PMC3795411

[B5] Craven L. Tuppen H. Greggains G. Harbottle S. Murphy J. Cree L. (2010). Pronuclear transfer in human embryos to prevent transmission of mitochondrial DNA disease. *Nature* 465 82–85. 10.1038/nature08958 20393463 PMC2875160

[B6] Dhillon V. Fenech M. (2014). Mutations that affect mitochondrial functions and their association with neurodegenerative diseases. *Mutat. Res. Rev. Mutat. Res.* 759 1–13. 10.1016/j.mrrev.2013.09.001 24055911

[B7] Elliott H. Samuels D. Eden J. Relton C. Chinnery P. (2008). Pathogenic mitochondrial DNA mutations are common in the general population. *Am. J. Hum. Genet.* 83 254–260. 10.1016/j.ajhg.2008.07.004 18674747 PMC2495064

[B8] Falk M. J. Decherney A. Kahn J. P. (2016). Mitochondrial replacement techniques–implications for the clinical community. *N. Engl. J. Med.* 374 1103–1106. 10.1056/nejmp1600893 26910290 PMC4936492

[B9] Fayzulin R. Perez M. Kozhukhar N. Spadafora D. Wilson G. Alexeyev M. (2015). A method for mutagenesis of mouse mtDNA and a resource of mouse mtDNA mutations for modeling human pathological conditions. *Nucleic Acids Res.* 43:e62. 10.1093/nar/gkv140 25820427 PMC4482060

[B10] Gammage P. Rorbach J. Vincent A. Rebar E. Minczuk M. (2014). Mitochondrially targeted ZFNs for selective degradation of pathogenic mitochondrial genomes bearing large-scale deletions or point mutations. *EMBO Mol. Med.* 6 458–466. 10.1002/emmm.201303672 24567072 PMC3992073

[B11] Gammage P. Van Haute L. Minczuk M. (2016). Engineered mtZFNs for manipulation of human mitochondrial DNA heteroplasmy. *Methods Mol. Biol.* 1351 145–162. 10.1007/978-1-4939-3040-1_11 26530680

[B12] Gammage P. A. Moraes C. T. Minczuk M. (2018). Mitochondrial genome engineering: the revolution may not be CRISPR-Ized. *Trends Genet.* 34 101–110. 10.1016/j.tig.2017.11.001 29179920 PMC5783712

[B13] Grady J. P. Pickett S. J. Ng Y. S. Alston C. L. Blakely E. L. Hardy S. A. (2018). mtDNA heteroplasmy level and copy number indicate disease burden in m.3243A>G mitochondrial disease. *EMBO Mol. Med.* 10:e8262. 10.15252/emmm.201708262 PMC5991564 29735722

[B14] Hashimoto M. Bacman S. Peralta S. Falk M. Chomyn A. Chan D. (2015). MitoTALEN: a general approach to reduce mutant mtDNA loads and restore oxidative phosphorylation function in mitochondrial diseases. *Mol. Ther.* 23 1592–1599. 10.1038/mt.2015.126 26159306 PMC4817924

[B15] Hirano M. Emmanuele V. Quinzii C. M. (2018). Emerging therapies for mitochondrial diseases. *Essays Biochem.* 62 467–481. 10.1042/ebc20170114 29980632 PMC6104515

[B16] Jo A. Ham S. Lee G. Lee Y. Kim S. Lee Y. (2015). Efficient mitochondrial genome editing by CRISPR/Cas9. *Biomed Res. Int.* 2015:305716. 10.1155/2015/305716 PMC4581504 26448933

[B17] Kang E. Wu J. Gutierrez N. Koski A. Tippner-Hedges R. Agaronyan K. (2016). Mitochondrial replacement in human oocytes carrying pathogenic mitochondrial DNA mutations. *Nature* 540 270–275. 27919073 10.1038/nature20592

[B18] Karicheva O. Z. Kolesnikova O. A. Schirtz T. Vysokikh M. Y. Mager-Heckel A. M. Lombes A. (2011). Correction of the consequences of mitochondrial 3243A>G mutation in the MT-TL1 gene causing the MELAS syndrome by tRNA import into mitochondria. *Nucleic Acids Res.* 39 8173–8186. 10.1093/nar/gkr546 21724600 PMC3185436

[B19] Kolesnikova O. A. Entelis N. S. Jacquin-Becker C. Goltzene F. Chrzanowska-Lightowlers Z. M. Lightowlers R. N. (2004). Nuclear DNA-encoded tRNAs targeted into mitochondria can rescue a mitochondrial DNA mutation associated with the MERRF syndrome in cultured human cells. *Hum. Mol. Genet.* 13 2519–2534. 10.1093/hmg/ddh267 15317755

[B20] Lightowlers R. Taylor R. Turnbull D. (2015). Mutations causing mitochondrial disease: what is new and what challenges remain. *Science* 349 1494–1499. 10.1126/science.aac7516 26404827

[B21] Loutre R. Heckel A. M. Jeandard D. Tarassov I. Entelis N. (2018a). Anti-replicative recombinant 5S rRNA molecules can modulate the mtDNA heteroplasmy in a glucose-dependent manner. *PLoS One* 13:e0199258. 10.1371/journal.pone.0199258 29912984 PMC6005506

[B22] Loutre R. Heckel A. M. Smirnova A. Entelis N. Tarassov I. (2018b). Can mitochondrial DNA be CRISPRized: Pro and Contra. *IUBMB Life* 70 1233–1239. 10.1002/iub.1919 30184317

[B23] Manwaring N. Jones M. Wang J. Rochtchina E. Howard C. Mitchell P. (2007). Population prevalence of the MELAS A3243G mutation. *Mitochondrion* 7 230–233. 10.1016/j.mito.2006.12.004 17300999

[B24] Mok B. Y. de Moraes M. H. Zeng J. Bosch D. E. Kotrys A. V. Raguram A. (2020). A bacterial cytidine deaminase toxin enables CRISPR-free mitochondrial base editing. *Nature* 583 631–637. 10.1038/s41586-020-2477-4 32641830 PMC7381381

[B25] Moretton A. Morel F. Macao B. Lachaume P. Ishak L. Lefebvre M. (2017). Selective mitochondrial DNA degradation following double-strand breaks. *PLoS One* 12:e0176795. 10.1371/journal.pone.0176795 28453550 PMC5409072

[B26] Patananan A. N. Wu T. H. Chiou P. Y. Teitell M. A. (2016). Modifying the mitochondrial genome. *Cell Metab.* 23 785–796. 10.1016/j.cmet.2016.04.004 27166943 PMC4864607

[B27] Peeva V. Blei D. Trombly G. Corsi S. Szukszto M. J. Rebelo-Guiomar P. (2018). Linear mitochondrial DNA is rapidly degraded by components of the replication machinery. *Nat. Commun.* 9:1727. 10.1038/s41467-018-04131-w PMC5928156 29712893

[B28] Phillips A. F. Millet A. R. Tigano M. Dubois S. M. Crimmins H. Babin L. (2017). Single-molecule analysis of mtDNA replication uncovers the basis of the common deletion. *Mol. Cell* 65 527–538.e6. 28111015 10.1016/j.molcel.2016.12.014

[B29] Reddy P. Ocampo A. Suzuki K. Luo J. Bacman S. Williams S. (2015). Selective elimination of mitochondrial mutations in the germline by genome editing. *Cell* 161 459–469. 10.1016/j.cell.2015.03.051 25910206 PMC4505837

[B30] Reddy P. Vilella F. Izpisua Belmonte J. C. Simon C. (2020). Use of customizable nucleases for gene editing and other novel applications. *Genes* 11:976. 10.3390/genes11090976 32842577 PMC7565838

[B31] Rhee W. J. Bao G. (2010). Slow non-specific accumulation of 2’-deoxy and 2’-O-methyl oligonucleotide probes at mitochondria in live cells. *Nucleic Acids Res.* 38:e109. 10.1093/nar/gkq050 20147460 PMC2875028

[B32] Sato R. Arai-Ichinoi N. Kikuchi A. Matsuhashi T. Numata-Uematsu Y. Uematsu M. (2018). Novel biallelic mutations in the PNPT1 gene encoding a mitochondrial-RNA-import protein PNPase cause delayed myelination. *Clin. Genet.* 93 242–247. 10.1111/cge.13068 28594066

[B33] Seibel P. Trappe J. Villani G. Klopstock T. Papa S. Reichmann H. (1995). Transfection of mitochondria: strategy towards a gene therapy of mitochondrial DNA diseases. *Nucleic Acids Res.* 23 10–17. 10.1093/nar/23.1.10 7870573 PMC306624

[B34] Shepherd D. L. Hathaway Q. A. Pinti M. V. Nichols C. E. Durr A. J. Sreekumar S. (2017). Exploring the mitochondrial microRNA import pathway through polynucleotide phosphorylase (PNPase). *J. Mol. Cell. Cardiol.* 110 15–25. 10.1016/j.yjmcc.2017.06.012 28709769 PMC5854179

[B35] Wallace D. (2010). Mitochondrial DNA mutations in disease and aging. *Environ. Mol. Mutagen.* 51 440–450. 20544884 10.1002/em.20586

[B36] Wallace D. Chalkia D. (2013). Mitochondrial DNA genetics and the heteroplasmy conundrum in evolution and disease. *Cold Spring Harb. Perspect. Biol.* 5:a021220. 10.1101/cshperspect.a021220 24186072 PMC3809581

[B37] Wang G. Chen H. Oktay Y. Zhang J. Allen E. Smith G. (2010). PNPASE regulates RNA import into mitochondria. *Cell* 142 456–467. 10.1016/j.cell.2010.06.035 20691904 PMC2921675

[B38] Wang G. Shimada E. Koehler C. M. Teitell M. A. (2012). PNPASE and RNA trafficking into mitochondria. *Biochim. Biophys. Acta* 1819 998–1007. 10.1016/j.bbagrm.2011.10.001 22023881 PMC3267854

[B39] Wang H. La Russa M. Qi L. (2016). CRISPR/Cas9 in genome editing and beyond. *Annu. Rev. Biochem.* 85 227–264. 27145843 10.1146/annurev-biochem-060815-014607 PMC13384723

[B40] Weninger A. Hatzl A. Schmid C. Vogl T. Glieder A. (2016). Combinatorial optimization of CRISPR/Cas9 expression enables precision genome engineering in the methylotrophic yeast *Pichia pastoris*. *J. Biotechnol.* 235 139–149. 10.1016/j.jbiotec.2016.03.027 27015975

[B41] Wolf D. Mitalipov N. Mitalipov S. (2015). Mitochondrial replacement therapy in reproductive medicine. *Trends Mol. Med.* 21 68–76. 10.1016/j.molmed.2014.12.001 25573721 PMC4377089

[B42] Yamada M. Emmanuele V. Sanchez-Quintero M. Sun B. Lallos G. Paull D. (2016). Genetic drift can compromise mitochondrial replacement by nuclear transfer in human oocytes. *Cell Stem Cell* 18 749–754. 10.1016/j.stem.2016.04.001 27212703 PMC9326498

[B43] Zetsche B. Gootenberg J. S. Abudayyeh O. O. Slaymaker I. M. Makarova K. S. Essletzbichler P. (2015). Cpf1 is a single RNA-guided endonuclease of a class 2 CRISPR-Cas system. *Cell* 163 759–771. 10.1016/j.cell.2015.09.038 26422227 PMC4638220

[B44] Zuker M. (2003). Mfold web server for nucleic acid folding and hybridization prediction. *Nucleic Acids Res.* 31 3406–3415. 10.1093/nar/gkg595 12824337 PMC169194

